# Multisite rate control analysis identifies ribosomal scanning as the sole high‐capacity/low‐flux‐control step in mRNA translation

**DOI:** 10.1111/febs.15059

**Published:** 2019-10-04

**Authors:** Helena Firczuk, James Teahan, Pedro Mendes, John E.G. McCarthy

**Affiliations:** ^1^ Warwick Integrative Synthetic Biology Centre [WISB] and School of Life Sciences University of Warwick Coventry UK; ^2^ Center for Quantitative Medicine UConn Health Farmington CT USA

**Keywords:** polypeptide initiation, protein synthesis, rate control, ribosomal scanning, yeast

## Abstract

Control of complex intracellular pathways such as protein synthesis is critical to organism survival, but is poorly understood. Translation of a reading frame in eukaryotic mRNA is preceded by a scanning process in which a subset of translation factors helps guide ribosomes to the start codon. Here, we perform comparative analysis of the control status of this scanning step that sits between recruitment of the small ribosomal subunit to the m^7^GpppG‐capped 5′end of mRNA and of the control exerted by downstream phases of polypeptide initiation, elongation and termination. We have utilized a detailed predictive model as guidance for designing quantitative experimental interrogation of control in the yeast translation initiation pathway. We have built a synthetic orthogonal copper‐responsive regulatory promoter (P_*CuR3*_) that is used here together with the *tet07* regulatory system in a novel dual‐site *in vivo* rate control analysis strategy. Combining this two‐site strategy with calibrated mass spectrometry to determine translation factor abundance values, we have tested model‐based predictions of rate control properties of the *in vivo* system. We conclude from the results that the components of the translation machinery that promote scanning collectively function as a low‐flux‐control system with a capacity to transfer ribosomes into the core process of polypeptide production that exceeds the respective capacities of the steps of polypeptide initiation, elongation and termination. In contrast, the step immediately prior to scanning, that is, ribosome recruitment via the mRNA 5′ cap‐binding complex, is a high‐flux‐control step.

## Graphical abstract

Translation of a reading frame on eukaryotic mRNA is preceded by a poorly understood scanning process in which a subset of translation factors helps guide ribosomes to the start codon. We use novel experimental and computational tools to study the control status of this scanning step that sits between recruitment of the small ribosomal subunit to the m^7^GpppG‐capped‐5′end of mRNA and the downstream phases of polypeptide initiation, elongation and termination.

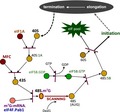

**Abbreviations**
CuRE: copper‐responsive regulatory element
DEAD: box proteins contain the motif Asp‐Glu‐Ala‐Asp
MFC: multifactor complex
$R_{1}^{J}$: response coefficient for protein synthesis as a function of intracellular translation factor abundance in the near‐physiological range
SBGN: Systems Biology Graphical Notation

## Introduction

Biological systems are generally highly complex and subject to multilayered control that can only be elucidated with the help of a combination of experimentation and computational modelling. The integration of multiple levels of system architecture generates higher order functionalities and/or emergent properties that cannot be deduced by simple extrapolation from the properties of the system components 1. A prime example of a complex biomolecular system is the protein synthesis machinery, which is ultimately responsible for creating all of the structures and functions that are associated with living cells 2, 3, 4. Maintaining an efficient, high‐precision mRNA translation machinery represents a major logistical and energetic burden for the cell, to the extent that, in the case of yeast, at least 76% of its total cellular energy budget is estimated to be committed to protein synthesis 5. In addition, this machinery needs to be capable of accurate regulatory responses to environmental change 6. At the heart of these key properties are features of control that are only beginning to be understood.

**Figure 1:**
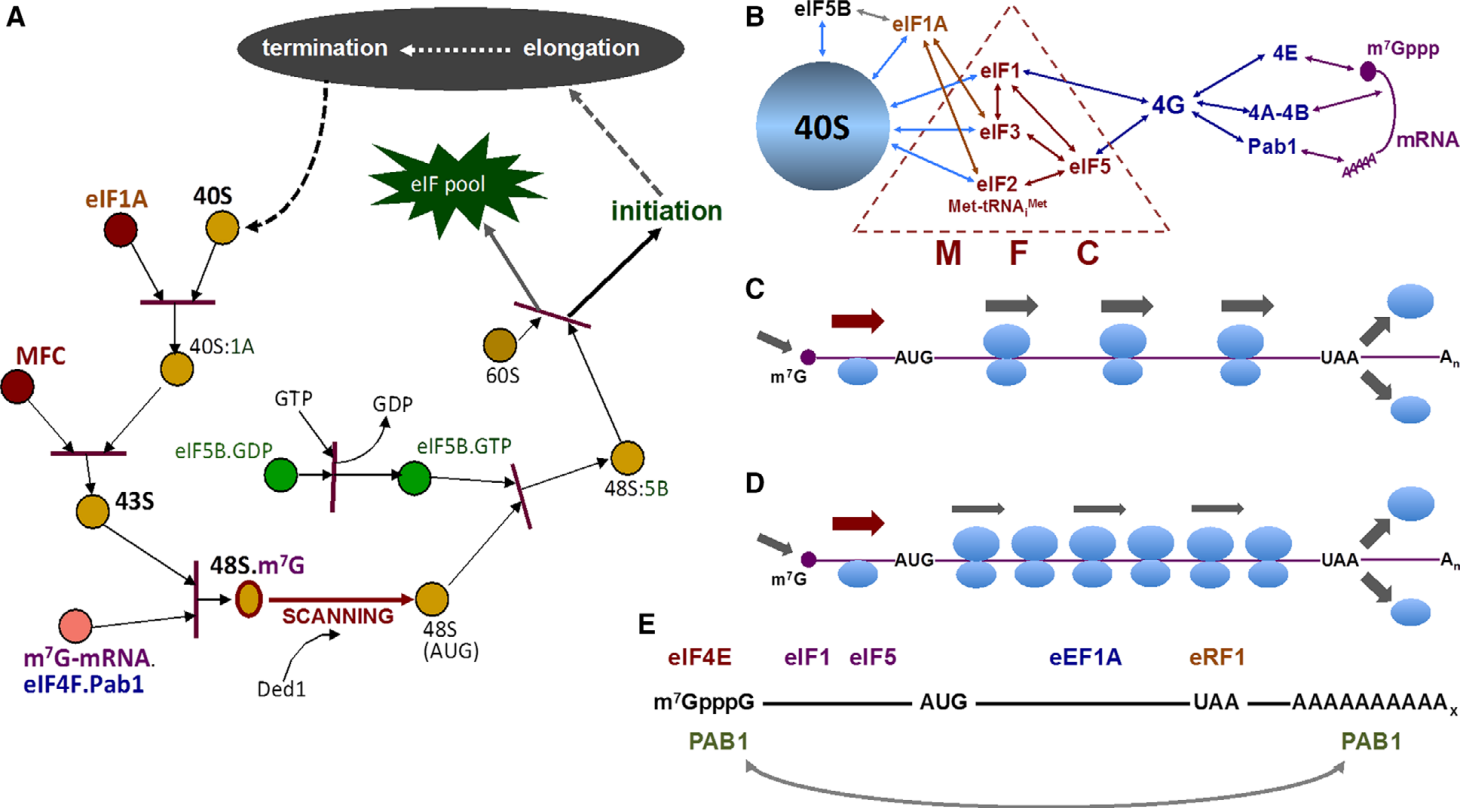
Molecular interactions and flows in eukaryotic translation. (A) Partial Petri net representation of translation scanning and initiation, in which the pathway is depicted as a series of bimolecular reactions/interactions. The scheme features the preformed MFC and cap‐binding complex bound to 5′‐capped mRNA (m^7^G‐mRNA.eIF4F.Pab1), whose component interactions are illustrated in panel B. Scanning is depicted as a fast step that transfers each ribosomal preinitiation complex (48S.m^7^G) from the 5′cap to the site of a start codon (48S.AUG) (panels A,C). Ded1 is thought to be a low‐flux‐control factor that has a more readily detectable influence on scanning efficiency along longer (structured) 5′UTRs 7. Under normal conditions, the elongation process is efficient, thus leaving sizeable gaps between elongating 80S complexes, while termination releases the separate ribosomal subunits back into the intracellular ribosome pool where they are again available for further initiation events (panel C). Attenuation of the rate of elongation, for example, caused by suppression of the activity of an elongation factor such as eEF1A, is expected to cause bunching up of the elongating 80S ribosomal complexes (panel D), thus retaining a greater proportion of the intracellular pool of ribosomal subunits associated with mRNP. Each pair of factors investigated in this study was selected from the set of translation factors indicated in panel E. These factors are respectively engaged in four steps: mRNA/ribosome recruitment (eIF4E), scanning (eIF1, eIF5), elongation (eEF1A), and termination (eRF1).

**Figure 2:**
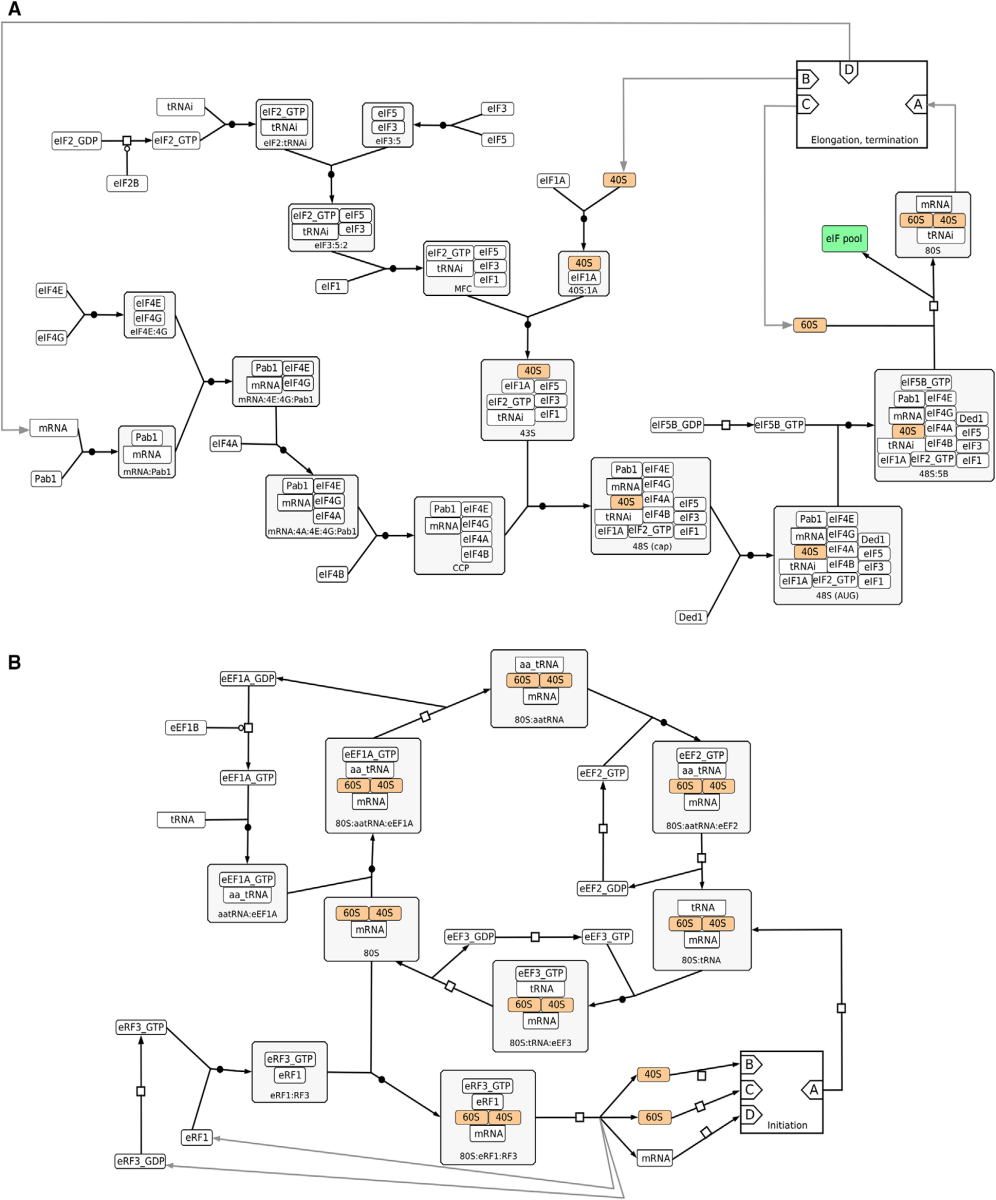
SBGN maps of the initiation (A) and elongation/termination (B) steps as represented in the computational model.

The translation pathway is thought to involve the progressive stoichiometric assembly (and disassembly) of multiple intermediate complexes (as shown for the scanning/initiation steps in Fig. 1A,B). Unexpectedly, we discovered previously that the intracellular abundance of the participating translation factors varies over at least a 20‐fold range 7, although the intersubunit stoichiometries in the complexes are generally unity (Fig. 1B). The exact number of formally recognized translation factors depends on the criteria used to define them, but it is generally agreed to be approximately 20 7. These proteins assist the ribosomes in multiple ways, manifesting a range of properties and functionalities, including: ATP/GTP hydrolysis or guanine nucleotide exchange 3, 8, 9, 10, remodelling of ribonucleoprotein complexes 11, 12, promoting specific intermolecular interactions (involving targets that include the m^7^Gppp cap 13, sites on the ribosome 2, tRNAs 2, 3 and other translation factors 14, 15), and molecular mimicry 15. Systems Biology Graphical Notation (SBGN 16) diagrams help to illustrate what we know about the roles of the respective factors and the relationships between them (Fig. 2). These diagrams also evince the complexity of a molecular machinery over which the cell must exercise precise control in order to ensure viability.

Eukaryotic translation depends on the recruitment of (5′‐capped) mRNA to the ribosomal 43S complex [comprising the 40S subunit plus the multifactor complex (MFC) factors eIF1, Met‐tRNA^Met^.eIF2.GTP, eIF3 and eIF5, together with eIF1A] in a step mediated by the cap‐binding complex, which in its minimal form comprises the cap‐binding proteins eIF4E and eIF4G 17. It is thought that the DEAD‐box helicase eIF4A is also part of the cap‐binding complex eIF4F, although it is, in itself, a poor RNA helicase that depends on interactions with other factors for its full functionality 18, 19. Moreover, interactions between eIF4G and the poly[A]binding protein Pab1 are capable of mediating interactions between the 5′ and 3′ ends of mRNA 20, whereby Pab1 stimulates both translation initiation 21, 22 and deadenylation by the Pan2/Pan3 complex 23. Scanning of the 5′ UTR by the 40S subunit is facilitated by translation factors that individually have been found to exercise very limited influence on rate control (these include eIF1, eIF3 and eIF5, all of which manifest very low steady‐state rate control [$R_{1}^{J}$ (response coefficient for protein synthesis as a function of intracellular translation factor abundance in the near‐physiological range)] values; 7). There is a further essential DEAD‐box helicase, called Ded1, that can associate with the cytoplasmic (and nuclear) cap‐binding complex 24, 25. This protein promotes the steps of scanning and polypeptide initiation, particularly on long 5′ UTRs, but the mechanism of its action is unclear 26. The progression of 40S ribosomal subunits along the mRNA during scanning is generally not dependent on specific recognition of nucleotides, and can be simulated using a partially random walk type of model 26. Specific recognition steps are, however, required to initiate scanning (5′ cap recognition mediated by eIF4E) and to enable polypeptide initiation (start codon recognition mediated by initiator‐tRNA in the ribosomal P‐site).

Once the polypeptide encoded by the main open reading frame has been initiated, the eukaryotic elongation factors take over. The elongation factor eEF1A delivers aminoacylated tRNAs to the ribosomal acceptor (A) site, while the eEF1B complex (comprising subunits α and β in yeast) promotes guanine nucleotide exchange on eEF1A 9. eEF2, on the other hand, is a GTP‐dependent translocase that is responsible for the movement of nascent peptidyl‐tRNAs from the A‐site to the P‐site on the ribosome 3. Deacylated tRNAs are released from the ribosomal exit (E) site in a process that in yeast (but not animals or plants) is promoted by eEF3 27. Uncharged tRNAs are recharged with the corresponding amino acids in preparation for another round of incorporation. Finally, polypeptide termination is triggered by the termination factor eRF1 upon recognition of a stop codon, whereby eRF1 is supported by eRF3, which has a ribosome‐dependent and eRF1‐dependent GTPase activity 15.

Given that protein synthesis is ultimately the source of all cellular structures and processes, and thus is of intrinsic importance to cell viability and selective competitiveness, research to characterize the principles of control in the translation machinery remains a major priority. We still do not understand, in precise terms, how interactions between the assemblage of translation machinery components determine the rate of protein synthesis, relationships that are of course fundamental to the regulatory responses of this system 28. A particularly distinctive feature of eukaryotic translation, compared to its prokaryotic counterpart, is the scanning process that links ribosomal recruitment of mRNAs via the 5′ end to polypeptide initiation at a start codon further along in the nucleotide sequence. Our earlier work 7 raised the possibility that the activities of the components supporting the scanning step in translation may be set at levels that could render their contributions less rate controlling than those of other factors. Clarification of rate control distribution in the translation machinery is critical to developing an understanding of the evolution of this important system. It is tempting to make *a priori* assumptions about the contributions of what are commonly referred to as ‘rate‐limiting’ steps to the overall control of translation. However, the nature of rate control in such a complex system can only be elucidated on the basis of quantitative experimental rate control analysis. Moreover, all of the translation factors act interactively as part of the overall translation machinery, and therefore it is essential that we examine the influence of combined multisite control modulations.

Here, we employ a novel dual‐site *in vivo* rate modulation strategy that has been designed to test the validity of hypotheses concerning control in such a complex molecular machinery. We use it to develop a wider picture of rate control in the scanning step as a whole, using yeast as a model system. This work also demonstrates that the combination of *in vivo* multisite rate control analysis with computational modelling is a broadly applicable strategy for elucidating control principles governing complex intracellular machineries, one that can be expected to contribute to the important wider goal of developing a meaningful *in silico* representation of at least the core processes of the living cell.

## Results

### Testable predictions of rate control based on a highly parameterized computational model

The complexity of the translation machinery makes it necessary to utilize computational modelling as a tool to help develop understanding of the rate control characteristics of this system. Our earlier work on the impact of changes in the abundance of individual translation factors on the translation process *in vivo* suggested that many of the factors associated with scanning exert minimal rate control when present at an abundance close to that of a wild‐type cell 7. This raises important questions about how scanning as an overall process contributes to the control of global protein synthesis. Here, we have utilized an established computational model 7 to provide more detailed (testable) predictions that can be used to help build a reliable picture of the distribution of control over the respective stages of protein synthesis. It is essential to use a model that is capable of reproducing the interdependence between the respective phases of translation. A key factor in determining our choice of this particular model is that it is highly detailed with regard to the mRNA recruitment and scanning steps. On the other hand, the coding region comprises a minimalized length of 20 codons, thus keeping calculation times within reasonable limits. Our strategy for using the model is to examine the predicted impact of the pairwise modulation of the intracellular abundance translation factors, since this represents a challenging test of the model's ability to simulate complex system behaviour. At the same time, it is important to note that this model was refined on the basis of fitting to single‐factor modulation data 7 and has been used here to provide indications of expected rate–activity relationships rather than accurate predictions of the results of dual‐factor modulation experiments.

**Figure 3:**
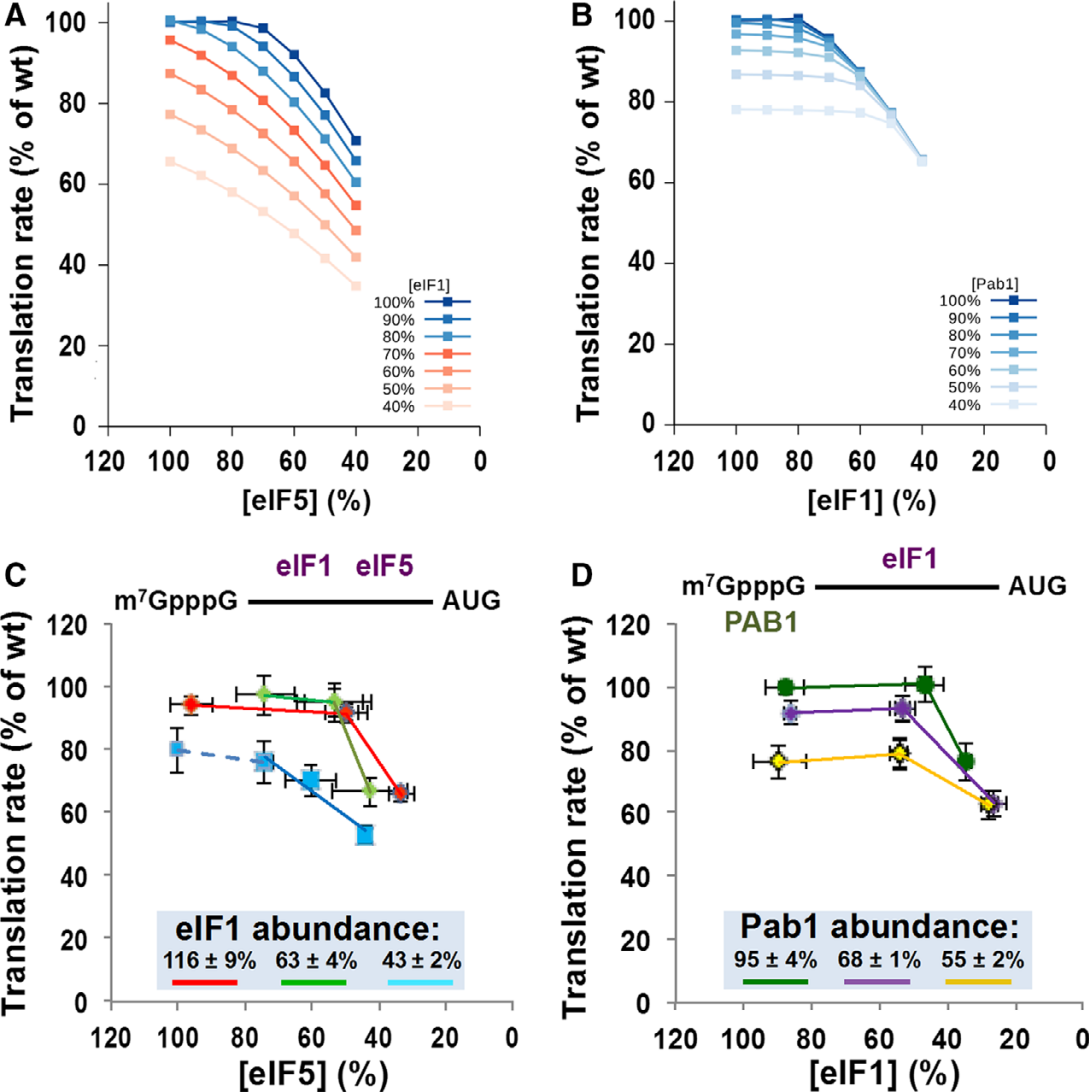
Predicted control characteristics for scanning factor pairs. The computational model predicts the outcomes of dual‐site regulatory regulation of pairs of translation factors: eIF1/eIF5 (panel A) and eIF1/Pab1 (panel B). For each pair, the expression of one translation factor gene is progressively suppressed against a background of different attenuated abundance levels of the second factor. A plateau of insensitivity is predicted in the near‐physiological region of factor abundance. In other words, as the abundance of the primary factor in each pair (e.g. eIF5 in panel A and eIF1 in panel B) is reduced progressively from the wild‐type abundance (100%), there is a zone in which the global translation rate remains unchanged. The curves manifesting this plateau‐type behaviour are coloured in blue. In panel A, the plateau is no longer evident in those curves generated at lower (below 80%) abundance values of the secondary factor (coloured in red). The corresponding experimental dual‐site control data are presented in panels C (eIF5/eIF1) and D (eIF1/Pab1). Expression of each of the genes encoding the respective translation factors was progressively and independently down‐regulated using genomic P_*CuR3*_ and P_*tetO7*_ regulatory promoter constructs. The abundance of each factor was determined using calibrated mass spectrometry (Fig. 8). In each case, the abundance of the ‘primary’ factor in the pair is plotted as a percentage of the wild‐type abundance on the *x*‐axis, while the three set levels of the secondary factor in each pair are given as abundance percentage values in the highlighted boxes within the plot areas.

**Figure 4:**
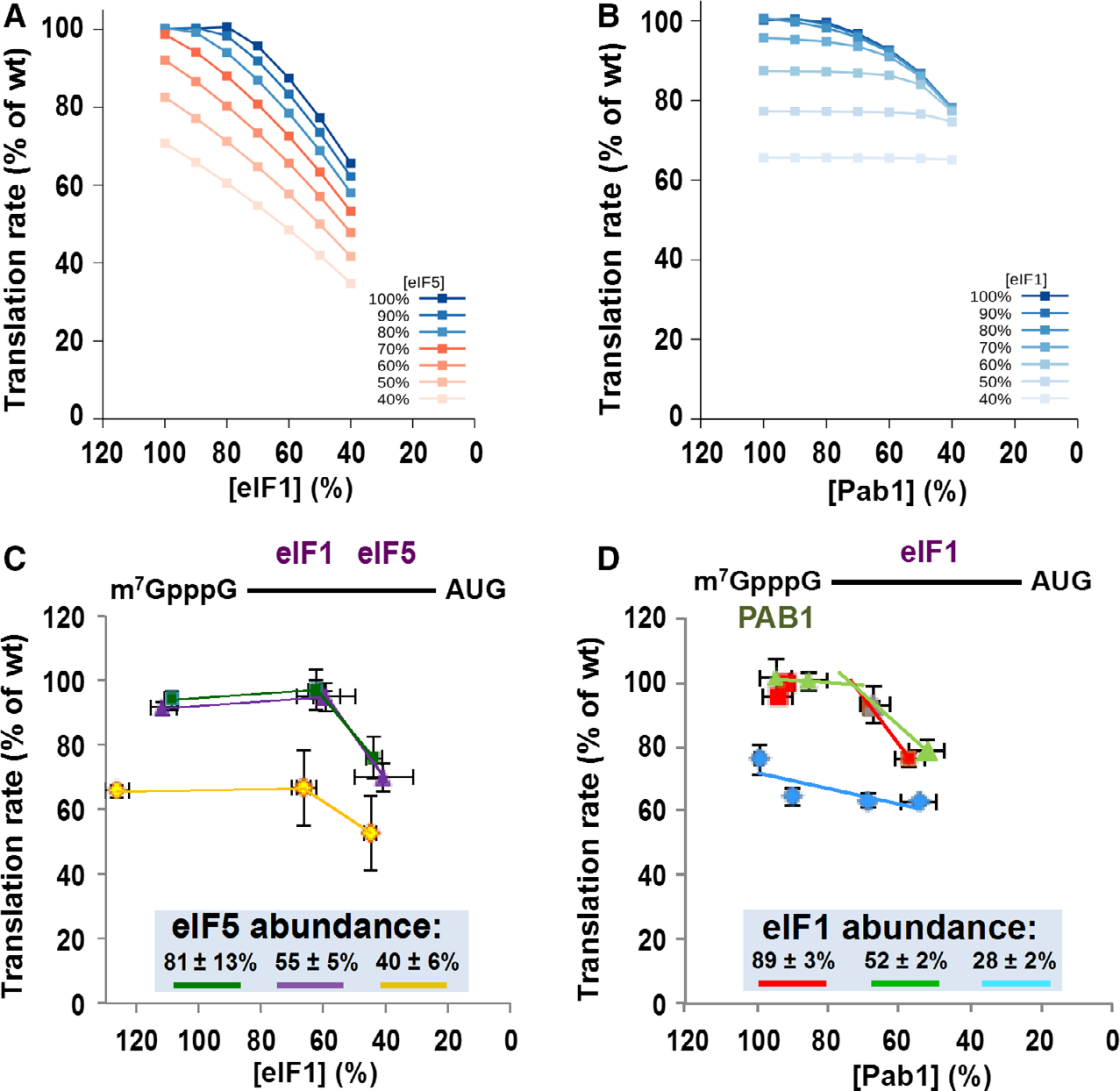
Mirror rate control plots to those shown in Fig. 3. The model output plot for the influence of variations in eIF1 abundance on global translation rate at different abundance levels of eIF5 (A) is compared to the equivalent experimental data (C). Similarly, the model (B) and experimental (D) plots are compared for the factor pair eIF1/Pab1.

This approach is exemplified by model outputs for the reciprocal relationship between the activities of eIF1 and eIF5 (Figs 3A and 4A). In each case, the translation rate is plotted against the intracellular abundance of one of the pair of factors over a range of different predetermined abundance values for the second factor. A striking feature of these model outcomes is the appearance of a plateau in the dependence of translation rate on abundance in the region near the physiological 100% (wild‐type) value. Such a plateau signifies marked insensitivity of the translation rate to changes in translation factor abundance, as would be expected if the factor has excess capacity in the near‐physiological concentration range. In the case of eIF1 and eIF5, reduction in the abundance of the second factor (e.g. eIF1 in Fig. 3A; eIF5 in Fig. 4A) leads to a progressive loss of the plateau. At even lower abundance values of the second factor (below approximately 80%), the ‘titrated’ first factor of the pair shows significant predicted rate control sensitivity at any abundance below 100% (see red lines in Figs 3A and 4A). The response relationship of translation rate to abundance changes for eIF1 and eIF5 in this region below 80% suggests that the contributions of eIF1 and eIF5 (to positioning of the initiator met‐tRNA in the ribosomal 40S subunit to enable successful scanning) are mutually additive.

We next compared the interdependence of rate control behaviour predicted for the translation factor pair eIF1 and Pab1. The latter protein plays a role in promoting recruitment of capped mRNAs (via the cap‐binding complex, and potentially also via the bridging complex between the cap‐binding complex and the mRNA 3′ end). In this case, the model predicts that both factors manifest very low rate sensitivity in the near‐physiological abundance range over a wide range of abundance values for the second factor in the pair. Indeed, the extent of the plateau increases as the abundance of the second factor (Pab1 in Fig. 3B; compare Pab1‐related rate sensitivity at different levels of eIF1 in Fig. 4B) is decreased. In other words, in marked contrast to the predicted relationship between eIF1 and eIF5, eIF1 and Pab1 are predicted to act upon the global translation rate via independent routes, whereby if one factor is subject to limitation this imposes a reduced minimum requirement (saturation threshold) to achieve maximal pathway flux for the other.

**Figure 5:**
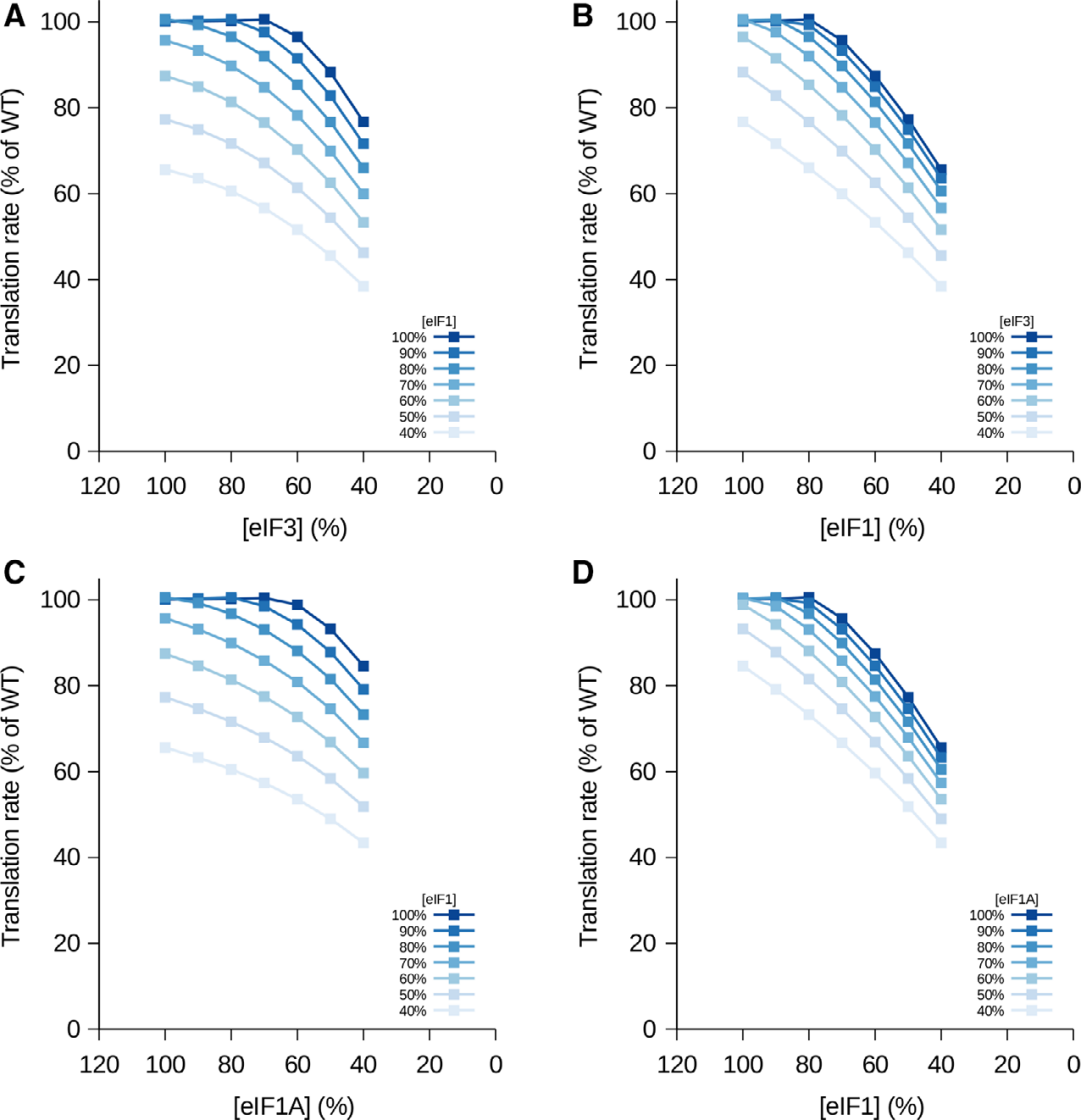
Dual‐site rate control plots generated by the computational model for translation initiation factors. Predicted relationships are shown for the translation factor pairs eIF3/eIF1 (A, B) and eIF1A/eIF1 (C, D).

We also performed modelling analysis of other scanning factor pairs in order to determine whether they are also predicted to manifest a similar pattern of minimal flux control in the near‐physiological range (Fig. 5). In the examples shown, we see that the outputs from the model for eIF3/eIF1 and eIF1A/eIF1 again predict pronounced rate insensitivity to variations in intracellular factor abundance at points close to 100% of the wild‐type level. Indeed, this distinctive rate control behaviour is generally predicted for the scanning translation factors, including those that comprise the MFC (Fig. 1B). In the next part of our work, we developed and implemented a novel experimental dual‐site ‘titration’ strategy that enables us to test such model‐derived predictions related to the interdependence of rate control by distinct factors.

### A synthetic dual‐site regulatory system for rate control analysis

**Figure 6:**
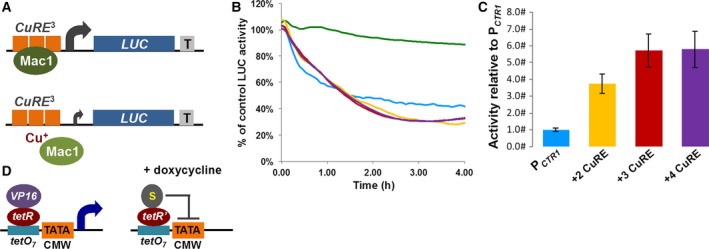
Regulatory promoters used for dual‐site modulation of the translation machinery. (A) The Mac1 transcription factor activates the promoter by binding to the CuRE, but its binding affinity is reduced in the presence of copper. The synthetic P_*CuR3*_ construct (A) was selected from a set of P_*CTR1*_ derivatives (panels B, C) in which we had inserted additional copies of the CuRE. The firefly luciferase (*LUC*) reporter gene was used to characterize the regulatory behaviour of the reporters. The repressibility of the three synthetic P_*CuR*_ promoters (B) was similar, but overall transcriptional activity was boosted by adding additional CuREs (C). Panels B and C share the same colour coding for the respective constructs; in addition, the green line in panel B records the activity generated by *LUC* transcribed from P_*CuR3*_ in the absence of added copper. P_*CuR3*_ was used in combination with the *tetO7* regulatory promoter (containing seven copies of the *tetO* box, which are bound by the doxycycline‐repressible *tetR*‐VP16 (tTA) hybrid transactivator 31; panel D), allowing us to simultaneously (but independently) down‐regulate the expression of a pair of translation factors in each experiment.

Rigorous *in vivo* experimental analysis of gene expression control requires suitably engineered orthogonally acting tools that work (progressively) within a suitable range. However, there is a marked paucity of negative regulatory promoters for use in yeast that can be precisely regulated and act orthogonally (i.e. in a way that does not interfere with metabolic or genetic processes that are not directly linked to the targeted gene). Therefore, for this study, we set out to develop a new synthetic regulatory promoter that could be applied in parallel to the *tet07* regulatory system (Fig. 6A–C). More specifically, we needed to be able to apply progressively variable modulation of the activities of pairs of translation factors, since this would facilitate direct testing of predictions derived from our computational model. The approach described here is in certain respects analogous to the systematic use of targeted dual‐gene mutation 29. However, our approach explores the more precisely controllable impact of the simultaneous progressive modulation of two gene expression rates rather than interactions between genetic modifications. Moreover, in designing our dual‐site regulatory system, we have ensured that progressive control can be applied in a way that allows us to study the effects of perturbations that impose only minimal deviations from the normal cellular state.

**Figure 7:**
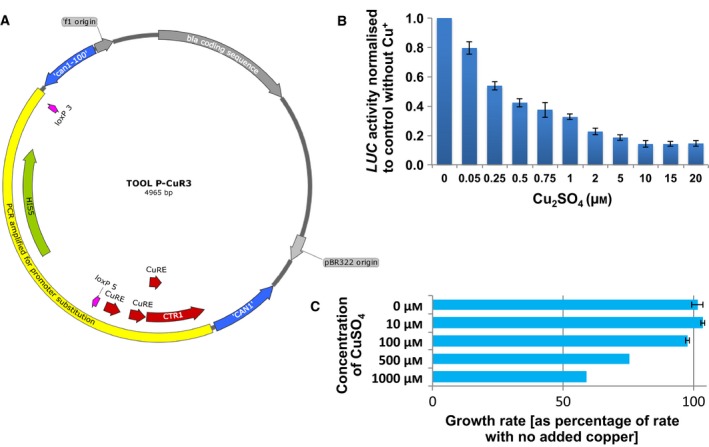
Repression behaviour of the synthetic P_*CuR3*_ promoter. (A) Map of the plasmid (TOOL‐P_*CuR3*_) bearing the synthetic P_*CuR3*_ promoter. (B) Progressive suppression of expression of the firefly *LUC* reporter transcribed from the P_*CuR3*_ promoter. The addition of copper sulphate to a final concentration of 10 μm results in maximal suppression. (C) The presence of 10 μm copper sulphate has no effect on growth.

The *tet07* regulatory system has proved to be a reliable orthogonal tool for analysis of rate control (7; Fig. 6D]. As the starting point for a second, complementary regulatory system, we utilized the yeast P_*CTR1*_ promoter, whose activity is modulated in response to changes in the concentration of copper 30. We constructed derivatives of this promoter in which we had inserted additional copper regulatory elements [CuRE (copper‐responsive regulatory element) elements; Fig. 6A]. We looked for a combination of dynamic range of regulation and maximum achievable level of transcription that would complement the regulatory characteristics of the *tet07* regulatory system 31. This was achieved using three CuRE elements (P_*CuR3*_), which maximized the nonrepressed activity of the promoter while maintaining the same dynamic range as wild‐type P_*CTR1*_. In further experiments, we established that the full dynamic range of the synthetic P_*CuR3*_ promoter could be explored using copper concentrations that were entirely nontoxic to *Saccharomyces cerevisiae* (Fig. 7). The addition of further CuRE sequences (as illustrated by P_*CuR4*_ in Fig. 6C) did not provide any improvement in terms of behavioural properties (Fig. 6B). We therefore used the synthetic promoter P_*CuR3*_ (Fig. 7A) in the dual‐site regulatory experiments described in this study.

### Dual‐site analysis of rate control in scanning

**Figure 8:**
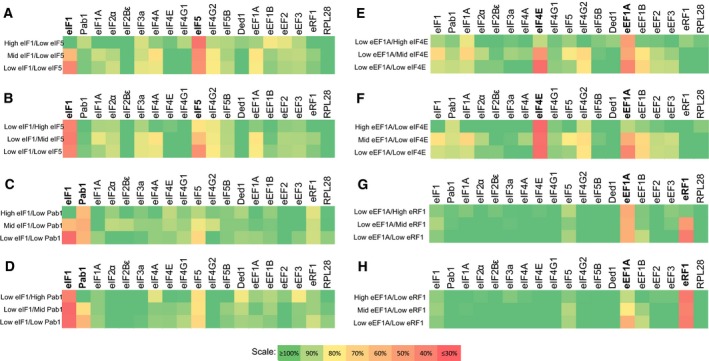
Mass spectrometric analysis of translation factor abundance values. Typical results of quantitative mass spectrometry (presented as heat maps) are shown for experimental dual rate control experiments involving modulation of eIF1/eIF5 (A, B), eIF1/Pab1 (C, D), eIF4E/eEF1A (E, F) and eEF1A/eRF1 (G, H). The heat maps show the abundance values for 19 translation factors in response to the presence of different concentrations of the regulatory ligands Cu^+^ and doxycycline. Each value represented in the heat maps is derived from at least three biological repeats. The relationship of the colour scale to the relative protein abundance is indicated at the bottom.

In reciprocal ‘genetic titration’ experiments, we have explored the rate control curves for eIF1 at different set abundances of eIF5, and vice versa. Both of these factors are involved in the scanning process 2. Importantly, quantification of the respective down‐regulated translation factors was achieved using standardized mass spectrometry in a strategy that allowed us to perform simultaneous control measurements on the other translation factors (Fig. 8A,B). The results demonstrate down‐regulation of translation factor activities (here evident as reduced protein abundance levels) corresponding to the genes placed under the control of the P_*CuR3*_‐ and *tetO7*‐regulated promoters. Taking into account the expected accuracy intrinsic to the mass spectrometric procedure, it is evident that the endogenous abundance values for the nonregulated factors were minimally affected. These results confirmed the specificity of the targeted regulatory changes brought about using genomic constructs transcribed from the P_*CuR3*_‐ and *tetO7*‐regulated promoters. At the same time we note that, as the expression of each gene encoding a translation factor is inhibited, global protein synthesis is, to differing degrees, also inhibited. Overall, therefore, in each experiment there is specific partial suppression of a selected translation factor relative to the other translation factors, accompanied by a reduction in the rate at which cells are formed. Further examples of the mass spectrometry outputs from other dual‐site analysis experiments are given in Fig. 8C–H.

In this context, it is important to note recent work indicating that eIF5 and eIF1 influence the stringency of start codon selection in mammalian cells 32. Discrimination against poor AUG context, albeit a relatively mild degree, has also been observed for eIF1 in yeast 33. This might explain the limited degree of interdependence between abundance we have observed for eIF1 and eIF5 (Fig. 8A,B). At the same time, the striking feature of our experimental data is that the $R_{1}^{J}$ value for eIF5 was unchanged within the 60–100% relative abundance range of eIF1, and slightly increased at 50% of the wild‐type eIF1 abundance (Fig. 3C), while, in the mirror experiment, the very low response coefficient ($R_{1}^{J}$) of eIF1 in the near‐physiological abundance range (80–100% of wild‐type abundance) of this factor remained unchanged at all concentration levels of eIF5 tested (from 40% up to 100% of the wild‐type abundance (Fig. 4C). We have calculated flux control coefficients for these respective dual modulation experiments and these are presented in the Supplementary Data section. If we now compare the experimental data with the predictions from the computational model (Figs 3A and 4A), we find that the model predicts low rate control sensitivity in the near‐physiological range for each of this pair of factors only in the presence of an abundance of the other factor that exceeds 80% of the physiological level. Below 80%, the model predicts full additivity between the rate control impacts of eIF1 and eIF5 (red lines in Figs 3A and 4A). Thus, the general form of the experimental curves is correctly predicted by the model, but the point at which increased rate control sensitivity for the ‘titrated’ factor in this pair becomes evident is shifted to a lower abundance level of the second factor.

Pab1 has been categorized as a multifunctional protein that is not dedicated to the translation process alone. It is not only thought to facilitate interactions between the 5′ and 3′ ends of mRNP molecules (via its interaction with eIF4G; 20,21) but is also believed to modulate deadenylation via its interactions with the Pan2‐Pan3 complex 23. Indeed, although reductions in the respective activities of eIF1 and eIF5 are tightly coupled with proportionate suppression of both global protein synthesis rate and growth, progressive diminution of Pab1 abundance has a more marked effect on growth than on global translation rate 7. However, Pab1 belongs to the group of low $R_{1}^{J}$ translation factors 7, and we sought to understand its role in terms of rate control in this context. We performed comparative experiments that explored the control relationship between Pab1 and eIF1 (Figs 3D and 4D). Once again, the minimal $R_{1}^{J}$ value of eIF1 was maintained over a wide range of Pab1 abundance values (100–50% of wild‐type abundance; Fig. 3D). In the mirror experiment, reductions in eIF1 abundance to 50% of the wild‐type abundance did not affect the very low $R_{1}^{J}$ value of Pab1 (Fig. 4D). Again, the computational model predicts (Figs 3B and 4B) the observed general form of the experimental curves for the eIF1/Pab1 pair.

### Rate control interactions across mRNA recruitment, elongation and termination

**Figure 9:**
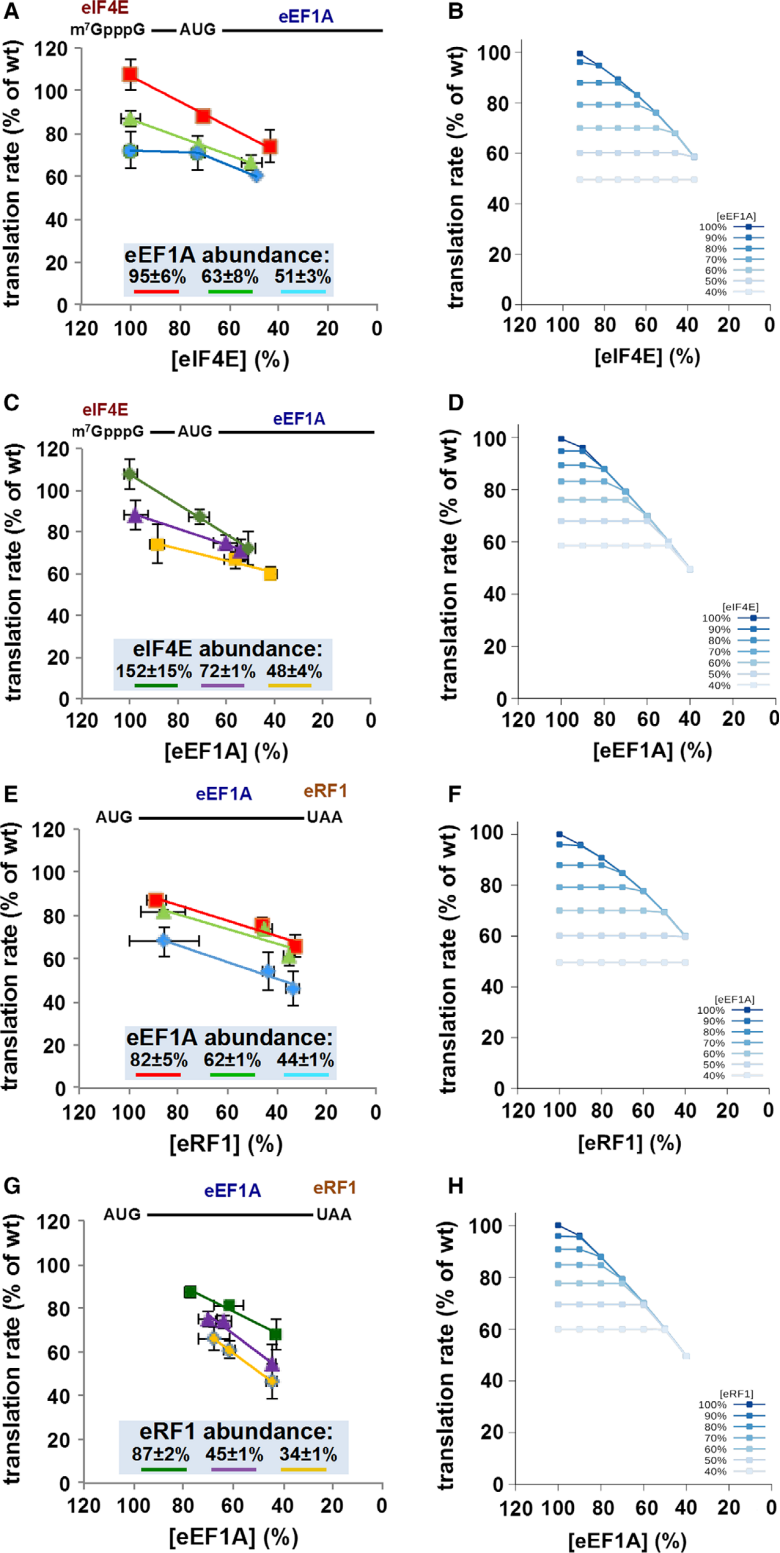
Dual‐site analysis targeted to high $R_{1}^{J}$ translation factors. Expression of each of the genes encoding the respective translation factors was progressively and independently down‐regulated using genomic P_*CuR3*_ and P_*tetO7*_ regulatory promoter constructs. The abundance of each factor was determined using calibrated mass spectrometry (Fig. 8). In each case, the abundance of the ‘primary’ factor in the pair is plotted as a percentage of the wild‐type abundance on the *x*‐axis, while the three set levels of the secondary factor in each pair are given as abundance percentage values in the highlighted boxes within the plot areas. Each experimental dual‐site rate control plot is paired with the equivalent computational model plot: eIF4E/eEF1A (A, B and C, D); eRF1/eEF1A (E, F and G, H).

For comparison, we extended our experimental analysis of the interfactor rate control relationships so that the overall study would include high‐flux‐control factors (with high response coefficients or $R_{1}^{J}$ values 7) that are involved in three of the four steps outlined in Fig. 1E: eIF4E (capped mRNA‐ribosome recruitment 17), eEF1A (elongation 9) and eRF1 (termination 15). The computational model has a reduced capability to predict the interdependence relationships involving elongation (or termination) because a minimal reading frame length is used that makes the model less well suited to simulating events on the longer reading frames that are typically found on eukaryotic mRNAs. This seems to be reflected in our comparative assessment of the modelling predictions with the experimental data (Fig. 9). The experimental data highlight the distinct rate control characteristics of steps outside of the scanning process (see also the flux control coefficients for the respective dual modulation experiments in the Supporting information). Down‐regulation of eIF4E against two reduced abundance levels of eEF1A was found to result in lessened responsiveness of translation rate in relation to eIF4E abundance (Fig. 9A), suggesting that the role of the cap‐binding protein in mRNA‐ribosome recruitment had become quantitatively less significant under conditions of constrained elongation. Examination of the model prediction for this relationship (Fig. 9B) reveals that this effect is captured by the modelling prediction, but that the model predicts a transition of the eIF4E $R_{1}^{J}$ value to zero at a higher concentration of eEF1A. In the mirror experiment (Fig. 9C), the eEF1A $R_{1}^{J}$ value dropped towards zero as the eIF4E abundance was reduced below 50%, suggesting a reciprocal interdependence of the rate control of the two factors. Here, the computational model predicted a transition of the eEF1A $R_{1}^{J}$ value to zero at comparatively high eIF4E abundance levels (Fig. 9D).

The experimental rate control interdependence plots for eEF1A and eRF1 are markedly different. We observed no significant changes in $R_{1}^{J}$ for eRF1 at reduced levels of eEF1A (Fig. 9E), whereas the $R_{1}^{J}$ value for eEF1A changed minimally at lower levels of eRF1 (Fig. 9G). The computational model, in contrast, predicted transitions to a zero $R_{1}^{J}$ value for the first titrated factor in each pair as the abundance of the second factor is reduced (Fig. 9F,H). Overall, we conclude that the experimental data reveal more complex behaviours that are readily distinguishable from the relationships that we find to be typical for the scanning‐related factors. At the same time, while correctly predicting high $R_{1}^{J}$ values for the individual factors eIF4E, eEF1A and eRF1, the computational model is less able to capture the observed interdependence of rate control for these factors.

## Discussion

Molecular systems biology, the combination of computational modelling with quantitative biochemical and biophysical analysis, is an essential platform for the elucidation of principles of control in complex biomolecular systems. Indeed, characterization of the quantitative principles of control operating in a biological system, like elucidation of structural and functional data on molecular components, is critical to a complete understanding of cell biology. In this study, we have examined the translation machinery, a highly complex system that, in one form or another, is at the heart of function and viability in all living organisms. The underpinning basis of control in such a system is not readily amenable to intuitive deduction, but here we present tools that provide valuable insight into fundamental control relationships between different steps on the protein synthesis pathway, thus enabling us to build a digital representation that will find broad application.

A computational model is only as valuable as the predictions it makes are verifiable. In this molecular systems biology approach, we have developed experimental tools that enable us to subject a highly detailed model of eukaryotic protein synthesis to validation. The observed lack of mutual influence of rate control behaviour (in the near‐physiological abundance range) for the two pairs of translation factors eIF1/eIF5 and eIF1/Pab1 confirms the validity of the model‐based prediction that the translation machinery is configured so as to minimize the impact of scanning on flux through the protein synthesis pathway. Moreover, Pab1, which interacts with both the poly[A] tail and the 5′ region of the mRNA (via the cap‐binding complex), is a low $R_{1}^{J}$ value multifunctional factor that also manifests minimal mutual influence over the control properties of other low $R_{1}^{J}$ value factors. Thus, in conclusion, the dual‐site analysis approach demonstrates that scanning is a low‐flux‐control phase that bridges two high‐flux‐control steps, that is, assembly of the cap‐binding complex on the 5′ end of the mRNA, and polypeptide elongation. At the same time, this study confirms the validity of the model prediction that combining low‐flux‐control steps imposes small flux changes in the overall pathway. We have therefore identified a novel collective property of the scanning‐promoting translation initiation factors that participate in this low‐flux‐control part of the translation pathway. This includes all of the MFC proteins (Fig. 1B), thus indicating that the MFC as a whole is a low‐flux‐control complex.

However, we also note that comparison of the modelling and experimental data reveals discrepancies under conditions of more extreme inhibition. For example, the experimental rate control behaviour (Figs 3C and 4C) observed for eIF1 and eIF5 deviates from the model predictions (Figs 3A and 4A) at more extreme degrees of limitation of the second factor abundance. These discrepancies are evident at factor abundance levels well below the physiologically normal intracellular levels, and we suspect that they occur because more complex behaviours begin to apply under conditions that become increasingly aberrant in relation to the normal growing cell. In the modelled scenarios in which we have changed the abundance levels of two factors, everything else has remained fixed. In a living cell, in contrast, major changes in the expression of even just one gene are likely to distort the expression of other genes. Under such conditions, a model that focuses only on one subcellular machinery becomes inadequate, especially where the imposed changes result in marked growth restriction. It is for this reason that *in vivo* rate control models are most useful when used to analyse the effects of (relatively small) parameter changes that do not result in major deviations from the standard physiological state of the cell. Over time, it may become possible to create (far more comprehensive) digital representations of global cellular activities that are capable of reflecting the complex effects that arise when intracellular processes are highly distorted.

Equally remarkable are the quite distinct experimentally determined rate control relationships for the paired high‐flux‐control factors that operate within the other steps in the translation pathway (Fig. 9). These confirm the status of capped mRNA‐ribosome recruitment, elongation and termination as steps of strong control in the translation machinery. As the abundance of eEF1A is reduced, the rate of elongation is constrained. As a result, it is expected that the throughput (rate of translocation) of elongating ribosomes on the mRNA population is attenuated, thus increasing the proportion (and mRNA packing density) of ribosomes actively engaged in elongation (Fig. 1C,D). This, in turn, is observed to reduce the maximum attainable number of initiations per unit time, most likely by virtue of the reduced size of the intracellular pool of ribosomal subunits. This is then reflected in a suppressed requirement for eIF4E‐mediated mRNA‐ribosome recruitment events (Fig. 9A). In the mirror experiment, we hypothesize that slowing eIF4E‐mediated mRNA‐ribosome recruitment limits the requirement for eEF1A‐promoted elongation cycles, possibly because there are fewer ribosomes actively elongating polypeptides on mRNA templates (Fig. 9C). The computational model is partially capable of capturing the observed transitions in $R_{1}^{J}$ values for these two factors.

On the other hand, the experimentally observed relationships between the activities of eEF1A and eRF1 are markedly different (Fig. 9E,G), and are likely to be affected by two factors. First, a slowing of the termination step directly influences the size of the pool of ribosomal subunits that are available for initiation by holding them up on the mRNA. Second, reductions in eRF1 abundance do not relate in a simple way to actual polypeptide terminations, because lower eRF1 activity is expected to enable, at least on some mRNAs, stop codon read‐through to lead to terminations at alternative stop codons further downstream rather than to simply block termination *per se* 34. It is difficult to characterize accurately the relative importance of the second effect, but it is likely to be less significant than that of the first point outlined above. In the case of the interdependence of rate control by eEF1A and eRF1, there are marked discrepancies between the experimental data and the model predictions. Apart from the limitations imposed by the use of a short reading frame in the model, it is important to point out that it also does not include steps that can reflect the effect of varying eRF1 abundance on translational read‐through.

Overall, these investigations show that scanning has evolved in eukaryotes as a highly efficient process that couples ribosome recruitment to polypeptide initiation, elongation and termination on each mRNA. We conclude that there is excess capacity in the scanning‐associated factors that renders the scanning process nonlimiting (due to abundance values in excess of requirements), thus limiting the impact of stochastic variations in scanning machinery capacity on global protein synthesis. This suggests that the cell expends a little extra energy in producing a small excess of the scanning‐related factors in order to prevent rate limitation at this nonsynthetic step that couples 40S‐mRNA recruitment with initiation at the start codon. Another aspect, which is beyond the scope of the present study, is that the factor requirements for scanning may change depending on the length of the 5′UTR. More specifically, it has been observed that the DEAD helicase Ded1 (and perhaps also Dbp1) exerts a particularly strong scanning‐promoting role in the case of long 5′UTRs 26. We believe that this aspect of the scanning process is worthy of further attention in future work.

This study illustrates how a molecular systems biology strategy can generate powerful insight into the quantitative rate control characteristics of a complex cellular pathway, insight that cannot be achieved by qualitative procedures. The utilization of calibrated quantitative mass spectrometry allows the comparative determination of the abundance levels across multiple components of the translation machinery, and is an approach that greatly enhances the analytical accuracy and power of rate control studies. It also highlights that the use of major disruptions of protein activity, whether caused by gene deletions or mutations or by large‐scale modulation of gene expression, is likely to provide a suboptimal basis for assessing the roles, particularly in terms of system control, of specific proteins in cellular machineries. This is because of the (often complex) collateral impact of disruptive changes on other cellular components. Minimally disruptive modulations that keep the cell close to its normal physiological state are more suitable for elucidating how a cellular machinery is controlled. As a consequence, the strategy we describe should be able to contribute to the ultimate digitization of cellular processes, a goal that must be at least partially attained if we are ever to approach an accurate (and predictive) understanding of the behavioural features that underpin the remarkable properties of living organisms. Progress in achieving this aim will of course depend upon further improvement of the computational models that are used in analysing system behaviour.

## Materials and methods

### Computational modelling

A computational model of translation that we developed previously 7 was the starting point for the modelling work described in this paper. The model is freely available from the BioModels database 35 with identifier BIOMD0000000457. Briefly, this is a differential equation‐based model describing initiation, elongation and termination. The model contains 156 distinct chemical species, 141 reactions and 56 rate constants. The steady‐state concentrations of the various proteins were determined by mass spectrometry, while the model parameters were calibrated by fitting to 212 distinct steady states obtained by titration of individual translation factor proteins 7. Here, we have simulated double‐modulation experiments using the parameter scan task in copasi, where the initial concentration of each protein of a pair is set to fractions of their steady‐state value in the original model (from 100% down to 40%). This generates predictions for the steady‐state translation rate of 49 distinct pairs of concentrations of the two proteins (while all other proteins are kept at their steady‐state concentrations). All computations were carried out using the software copasi 36, 37 version 4.24.

### Strain construction

Strains used in this study were all derived from the background strain PTC41: *MATα ade2‐1 ura3‐1 leu2‐3,112 his3‐11,15 can1‐100* (a derivative of W303). Promoters (P_*tetO7*_ with *kanMX* and P_*CuR3*_ with *HIS5* marker) were PCR amplified from the vectors pCM225 31 and TOOL‐P_*CuR3*_ (created for this study; Fig. 7 and Supporting information), respectively, using primers that include sequences homologous to target promoter regions to enable substitution of the region −60 to −1 upstream of each translation factor CDS with one of the regulatable promoter/5′UTR cassettes. After integration, the *HIS5* marker was removed from the P_*CuR3*_ promoter; it could therefore be used independently for the integration of ‘top‐up’ constructs, which served to help adjust selected translation factor levels to the required levels 7. Expression ‘top‐up’ constructs were genomically integrated using either the *HIS5* marker targeted to the *can1* locus or the *BLE* (phleomycin resistance) marker targeted to the *lys2* locus . For the eEF1A strain, top‐up expression was achieved by substituting the natural P_*TEF2*_ promoter with the P_*HYP2*_ promoter. A full strains table is provided in the Supporting information.

### Dual‐site rate control experiments

In order to accurately determine growth rate [in YNBD‐Met(‐Ura) medium], protein synthesis rate (by ^35^S‐l‐methionine incorporation) and relative translation factor abundance (using mass spectroscopy), a strict 3‐day experimental routine was followed. Each set of cultures included two independent PTC41 control cultures and measurements involved up to 10 different test conditions. On the first day, overnight cultures (of PTC41 and of the test strain) in 10 mL of YNBD‐Met(‐Ura) was inoculated with single colonies from plates no more than 3 weeks old. The next morning, these cultures were diluted to OD_600_ = 0.2 in 10 mL of YNBD‐Met(‐Ura), grown for 5–6 h to reach exponential phase and then diluted to OD_600_ = 0.004–0.02 in 20 mL of YNBD‐Met(‐Ura) (depending on expected growth rates; the slower the growth, the higher the starting OD_600_ set by dilution), followed by overnight growth for 17 h (to OD_600_ of approximately 1.2).

At this stage, preselected doxycycline and copper concentrations were established in each culture. Exploratory experiments were performed in order to identify the concentrations of these regulatory ligands that would enable us to cover the required range of translation factor abundances, and thus translation rates. Seventeen hours of further growth in the presence of doxycycline and copper ensured that the inhibitory effect on transcription of the targeted translation factor gene was stably reflected in steady‐state mRNA and encoded protein levels. On the third day, the cultures were diluted again in 20 mL of YNBD‐Met(‐Ura), maintaining the same doxycycline and copper concentrations, to OD_600_ = 0.10–0.25. Only cultures that had similar OD_600_ values to the PTC41 reference strain were diluted and used for further experiments. To determine exponential growth rates, the optical density was monitored over the following 4.5 h until the cultures reached OD_600_ = 0.5. At this point, samples for western blotting were collected (an equivalent of 10 mL of cells at OD_600_ = 0.5), and the cultures were diluted to OD_600_ = 0.1 in 10 mL of YNBD‐Met(‐Ura) (again, only the cultures with the same optical density as PTC41 were processed). After a further 15 min of growth, 100 μL of labelling mix (0.38 MBq of ^35^S‐l‐methionine in 2 μg·mL^−1^ methionine) was added to the each culture and samples were collected every 3 min (over the next 12 min; five samples in total per culture). The proteins in each sample were precipitated by TCA and the amount of radioactivity incorporated into proteins was measured using a scintillation counter 38. In all cases there was a linear accumulation of radioactivity over time, and the slope was used to calculate the relative protein synthesis rate in relation to PTC41.

### Mass spectrometry

Each strain was grown in triplicate, using the same 3‐day growth protocol as for the protein synthesis/growth rate measurements. For each experiment, a 20‐mL yeast culture was incubated with shaking at 30 °C until OD_600_ = 0.5 was reached. After centrifugation and resuspension in 50 mm NH_4_HCO_3_, 15 mL of each culture was then transferred to a bead‐beater tube and stored at −20 °C. Subsequently, after thawing, glass beads were added in 50 μL of 50 mm NH_4_HCO_3_ and the tubes were shaken in the bead beater (10 × 1 min shaking periods, with 2‐min breaks in between). The tubes were then pierced with a hot needle and centrifuged so that the lysate could be collected and placed into low‐bind Eppendorf tubes. A 10‐μL sample was taken from each tube for measurement of the lysate concentration using a NanoDrop spectrophotometer (ThermoFisher Scientific, Waltham, MA, USA). Lysates were stored at −20 °C until trypsin digestion was performed, and subsequently transferred to −80 °C for long‐term storage. For digestion, 1.1 mg of lysate (equivalent to approximately 60 million cells) was incubated with trypsin (according to a previously described protocol 39). Digested samples were stored at −20 °C until prepared for mass spectrometry; this preparation involved mixing 12 μL of each digest, 100 μL of a mixture of peptide standards, comprising 2.5 nm GluFib peptide (F3261; Sigma Aldrich, Dorset, England) and ^13^C‐L‐Arg/^13^C‐L‐Lys‐labelled, trypsin‐digested Ribo3 QconCAT protein (comprising peptides corresponding to multiple translation factors, as described in reference 7), and 88 μL of NH_4_HCO_3_ buffer. Mass spectrometry measurements were performed on a Thermo Scientific™ TSQ Quantiva™ Triple Quadrupole Mass Spectrometer with an UltiMate 3000 RSLCnano System (Thermo Scientific). Data analysis was performed using skyline software 40. The relative protein concentration was determined by dividing each abundance value by the reference value obtained for PTC41 (Supporting information).

## Conflicts of interest

The authors declare no conflict of interest.

## Author contributions

This study was designed by JEGM and PM. Experiments were performed by HF and JT; computational modelling was performed by PM. The manuscript was written by JEGM.

## Supporting information


**Appendix S1.** Description of procedure to estimate protein abundance values.
**Table S1**. *S. cerevisiae* strains including expression top‐up plasmids
**Fig. S2.** Complete nucleotide sequence of plasmid carrying the synthetic regulatable P_*CuR3*_ promoter
**Appendix S2.** Estimated $R_{1}^{J}$ values derived from the experimental dual‐site rate control data.
