# Supplementary material for: Multisite rate control analysis identifies ribosomal scanning as the sole high‐capacity/low‐flux‐control step in mRNA translation
Source: FEBS J. 2019 Oct 4;287(5):925–40. doi: 10.1111/febs.15059 (PMC7054134; doi:10.1111/febs.15059)
Supplement: Supplementary file 1 — Appendix S1. Description of procedure to estimate protein abundance values. Table S1. S. cerevisiae strains including expression top‐up plasmids Fig. S2. Complete nucleotide sequence of plasmid carrying the synthetic regulatable PCuR3 promoter Appendix S2. Estimated R1J values derived from the experimental dual‐site rate control data. [file FEBS-287-925-s001.zip › febs15059-sup-0001-Supinfo.pdf]

FEBS PRESS
science publishing by scientists
WILEY
The FEBS Journal
www.febsjournal.org

Multisite rate control analysis identifies ribosomal scanning as the sole high-capacity/low-flux-control step in mRNA translation

Helena Firczuk, James Teahan, Pedro Mendes and John E.G. McCarthy

DOI: 10.1111/febs.15059

Supporting Information

Using MS data to estimate protein abundance values

Three biological repeats were collected for each test condition. Given that there were 9 conditions for each of the four translation factor pairs, this yielded 108 samples to be analyzed using mass spectrometry. In addition, we performed 12 biological repeats of the wild-type PTC41 strain, and these samples were evenly spread between the other experimental runs. After all of the data had been collected, the results were imported into Skyline software for initial analysis.

All of the transition peaks were viewed, and the peak positions were manually corrected if the automated peak assignment was clearly misaligned. If there was no obvious solution to misaligned peaks the transitions were marked as unreliable and removed from the analysis. In this way, the total number of transitions used in this analysis was reduced from 959 to 527. The data of signal intensities of all the transitions was then exported for further analysis in Microsoft Office Excel.

The following calculations were performed for each sample:

1. The peptide signal intensity was calculated as a sum of signals from all of the transitions measured for this peptide.
2. The protein signal intensity was then calculated as a sum of signals from all of the peptides measured for a given protein.
3. The loading control intensity was calculated as a geometric mean of signals for: unlabelled GluFib peptide and labelled GluFib and Fib peptides from the Ribo3 QconCAT protein*.
4. The loading-corrected protein signal was calculated by dividing the protein signal intensity (p2) by the intensity of the loading control (p3), thus correcting the protein signal for the differences in loading.
5. The weighted calibrator signal intensity was calculated as the geometric mean of signal of the Rps5, Rpl28 and Act1 proteins.
6. The calibrated protein signal was calculated by dividing the loading-corrected protein signal (p4) by the weighted calibrator signal intensity (p5).
7. If there was a labelled reference peptide present for a given protein – it was treated in the same way as other signals, subject to calculation of the heavy to light ratio.
8. The average was calculated for the data from the biological repeats of the same condition.
9. The relative protein concentration was calculated by dividing the average value obtained for each protein by the average value obtained for the PTC41 reference strain.

* Firczuk,H., Kannambath,S., Pahle,J., Claydon,A., Beynon,R., Duncan,J., Westerhoff,H., Mendes,P. and McCarthy,J.E.G. (2013) An *in vivo* control map for the eukaryotic mRNA translation machinery. *Mol. Sys. Biol.,* **9**, 635.

TOOL-P_{CuR3}

Complete nucleotide sequence of plasmid shown in Supplementary Figure S2, with synthetic P_{CuR3} sequence highlighted in red.

TTGGGTGCACGAGTGGGTTACATCGAACTGGATCTCAACAGCGGTAAGATCCTTGAGAGTTTTCGCCCCGAA
GAACGTTTTCCAATGATGAGCACTTTTAAAGTTCTGCTATGTGGCGCGGTATTATCCCGTATTGACGCCGGG
CAAGAGCAACTCGGTCGCCGCATACACTATTCTCAGAATGACTTGGTTGAGTACTCACCAGTCACAGAAAAG
CATCTTACGGATGGCATGACAGTAAGAGAATTATGCAGTGCTGCCATAACCATGAGTGATAACACTGCGGCC
AACTTACTTCTGACAACGATCGGAGGACCGAAGGAGCTAACCGCTTTTTTGCACAACATGGGGGATCATGTA
ACTCGCCTTGATCGTTGGGAACCGGAGCTGAATGAAGCCATACCAAACGACGAGCGTGACACCACGATGCCT
GCAGCAATGGCAACAACGTTGCGCAAACTATTAACTGGCGAACTACTTACTCTAGCTTCCCGGCAACAATTA
ATAGACTGGATGGAGGCGGATAAAGTTGCAGGACCACTTCTGCGCTCGGCCCTTCCGGCTGGCTGGTTTATT
GCTGATAAATCTGGAGCCGGTGAGCGTGGGTCTCGCGGTATCATTGCAGCACTGGGGCCAGATGGTAAGCCC
TCCCGTATCGTAGTTATCTACACGACGGGGAGTCAGGCAACTATGGATGAACGAAATAGACAGATCGCTGAG
ATAGGTGCCTCACTGATTAAGCATTGGTAACTGTCAGACCAAGTTTACTCATATATACTTTAGATTGATTTA
AAACTTCATTTTTAATTTAAAAGGATCTAGGTGAAGATCCTTTTTGATAATCTCATGACCAAAATCCCTTAA
CGTGAGTTTTCGTTCCACTGAGCGTCAGACCCCGTAGAAAAGATCAAAGGATCTTCTTGAGATCCTTTTTTT
CTGCGCGTAATCTGCTGCTTGCAAACAAAAAAACCACCGCTACCAGCGGTGGTTTGTTTGCCGGATCAAGAG
CTACCAACTCTTTTTCCGAAGGTAACTGGCTTCAGCAGAGCGCAGATACCAAATACTGTCCTTCTAGTGTAG
CCGTAGTTAGGCCACCACTTCAAGAACTCTGTAGCACCGCCTACATACCTCGCTCTGCTAATCCTGTTACCA
GTGGCTGCTGCCAGTGGCGATAAGTCGTGTCTTACCGGGTTGGACTCAAGACGATAGTTACCGGATAAGGCG
CAGCGGTCGGGCTGAACGGGGGGTTCGTGCACACAGCCCAGCTTGGAGCGAACGACCTACACCGAACTGAGA
TACCTACAGCGTGAGCTATGAGAAAGCGCCACGCTTCCCGAAGGGAGAAAGGCGGACAGGTATCCGGTAAGC
GGCAGGGTCGGAACAGGAGAGCGCACGAGGGAGCTTCCAGGGGGAAACGCCTGGTATCTTTATAGTCCTGTC
GGGTTTCGCCACCTCTGACTTGAGCGTCGATTTTTGTGATGCTCGTCAGGGGGGCGGAGCCTATGGAAAAAC
GCCAGCAACGCGGCCTTTTTACGGTTCCTGGCCTTTTGCTGGCCTTTTGCTCACATGTTCTTTCCTGCGTTA
TCCCCTGATTCTGTGGATAACCGTATTACCGCCTTTGAGTGAGCTGATACCGCTCGCCGCAGCCGAACGACC
GAGCGCAGCGAGTCAGTGAGCGAGGAAGCGGAAGAGCGCCTGATGCGGTATTTTCTCCTTACGCATCTGTGC
GGTATTTCACACCGCATATATGGTGCACTCTCAGTACAATCTGCTCTGATGCCGCATAGTTAAGCCAGTATA
CGACGTCAAGCCAAAGCGCCAAATGCAGCAGTAACGAAAACTGCAATGTATGGAACACCACCTTTGGTGGTC
CTTGACAGGAATTTAGGAGCCAACTTGTTCTTTGATAGACCAAATAAAATACGGGAACCAACGTAAATATTT
GAATTTGCGGCAGAAATAATGGTTGTTAAGATAACAGCGTTGAAGATATGTGGCAAAACCTTTGTACCAGAG
TTCTCAATAGCAATAATAAAGGGAGAAGTAGAAACGTAGGAAGTAGATTGTGTTAGTTTAGGGTCATTGTAT
GGAACTAAAAGTCCAATGAATAATAGAGAGCCAATGTAGAAGGTTAAGATACGGAAAACAACTTTTTTGATG
GCTCTTGGAACGGATTTTCTGAGGCCTTGCAAACTAGTGGCGCCATTTTGAATGTCAAATATAATACACTTT
TTTTTATTTTCTATTCGATTCTTCCAACAAATGTAATTTGAGTCGCCGCCGAACTATTACTCCACCTGTTTA
AGTTTGTGCTTAAAAGTTTTGTTATGCAAATAAAGCTATGGAGTAGTTGTTTATTAAGAGCACACTGCGTAT
CTGTTTTACTAATGTGAACTAATCATAAGAGGAATAAAACATACAAGACCCTCTCGAGATGACAATACAACA
TTTTTTATGTTGCATTTTGTAGCCCGAATCTTGAAAAGTGCTCTTTTCAGGATCGTGCCATTTTTGCTCATT
TTACCGTCGTCTTGAGCAAATATCCCATGATTTGCATCATATGCATTTTGTAGCCCGAATCTTGAAAAGTGC
TCTTTTCAGGATCGTGCCATTTTTGCTCATTTTACCGTCGTCTTGAGCAAATATCCCATGATTTGCATCAAT
ACATGAGAACATAACCACAGTGTCGACCCGCGGAAAAATTCGAGTTTTATGTTGCATTTTGTAGCCCGAATC
TTGAAAAGTGCTCTTTTCAGGATCGTGCCATTTTTGCTCATTTTACCGTCGTCTTGAGCAAATATCCCATGA
TTTGCATCAATACATGAGAACGTCGACCCGCGGCATATGCTCGACAACCCTTAATATAACTTCGTATAATGT
ATGCTATACGAAGTTATTAGGTCTAGAGATCTGTTTAGCTTGCCTCGTCCCCGCCGGGTCACCCGGCCAGCG
ACATGGAGGCCCAGAATACCCTCCTTGACAGTCTTGACGTGCGCAGCTCAGGGGCATGATGTGACTGTCGCC
CGTACATTTAGCCCATACATCCCCATGTATAATCATTTGCATCCATACATTTTGATGGCCGCACGGCGCGAA
GCAAAAATTACGGCTCCTCGCTGCAGACCTGCGAGCAGGGAAACGCTCCCCTCACAGACGCGTTGAATTGTC
CCCACGCCGCGCCCCTGTAGAGAAATATAAAAGGTTAGGATTTGCCACTGAGGTTCTTCTTTCATATACTTC
CTTTTAAAATCTTGCTAGGATACAGTTCTCACATCACATCCGAACATAAACAACCATGGGTAGGAGGGCTTT
TGTAGAAAGAAATACGAACGAAACGAAAATCAGCGTTGCCATCGCTTTGGACAAAGCTCCCTTACCTGAAGA
GTCGAATTTTATTGATGAACTTATAACTTCCAAGCATGCAAACCAAAAGGGAGAACAAGTAATCCAAGTAGA
CACGGGAATTGGATTCTTGGATCACATGTATCATGCACTGGCTAAACATGCAGGCTGGAGCTTACGACTTTA
CTCAAGAGGTGATTTAATCATCGATGATCATCACACTGCAGAAGATACTGCTATTGCACTTGGTATTGCATT
CAAGCAGGCTATGGGTAACTTTGCCGGCGTTAAAAGATTTGGACATGCTTATTGTCCACTTGACGAAGCTCT
TTCTAGAAGCGTAGTTGACTTGTCGGGACGGCCCTATGCTGTTATCGATTTGGGATTAAAGCGTGAAAAGGT

TGGGGAATTGTCCTGTGAAATGATCCCTCACTTACTATATTCCTTTTCGGTAGCAGCTGGAATTACTTTGCA
TGTTACCTGCTTATATGGTAGTAATGACCATCATCGTGCTGAAAGCGCTTTTAAATCTCTGGCTGTTGCCAT
GCGCGCGGCTACTAGTCTTACTGGAAGTTCTGAAGTCCCAAGCACGAAGGGAGTGTTGTAAAGAGTACTGAC
AATAAAAAGATTCTTGTTTTCAAGAACTTGTCATTTGTATAGTTTTTTTATATTGTAGTTGTTCTATTTTAA
TCAAATGTTAGCGTGATTTATATTTTTTTTCGCCTCGACATCATCTGCCCAGATGCGAAGTTAAGTGCGCAG
AAAGTAATATCATGCGTCAATCGTATGTGAATGCTGGTCGCTATACTGCTGTCGATTCGATACTAACGCCGC
CATCCAGTTTAAACGAGCTCTCGAGAACCCTTAATATAACTTCGTATAATGTATGCTATACGAAGTTATTAG
GTGATATCGGATCCAAGCTTCAGCTGAGCTCAGATCTTTCTGCGGCCGCCAGTGGAACTTTGTACGTCCAAA
ATTGAATGACTTGGCCAACTACACTAAGTTCCAGGGCAAAAGTGATTGCCCAAGAAAACCAATACATGTAAC
CATTGGCCGCACCAAATGCTGGAGAAAGGAATCTTTGTGAGAAAACTGTGAAAGAGGATGTAACAGGGATGA
ATGTAGCCATTTCACCCAAGGACTGCGTGACAGAATATGCCAAAGAACCCATAAATAAATATGATATAAGAG
CGCCCACTGGGCCGGCGTTGGTCAGAGGTGTGGATAAACCAATGAAAAGACCTGTACCAATAGTACCACCAA
GGGCAATCATACCGGTTATAAGGGATTTTGCCGATTTCGGCCTATTGGTTAAAAAATGAGCTGATTTAACAA
AAATTTAACGCGAATTTTAACAAAATATTAACGTTTACAATTTCAGGTGGCACTTTTCGGGGAAATGTGCGC
GGAACCCCTATTTGTTTATTTTTCTAAATACATTCAAATATGTATCCGCTCATGAGACAATAACCCTGATAA
ATGCTTCAATAATATTGAAAAAGGAAGAGTATGAGTATTCAACATTTCCGTGTCGCCCTTATTCCCTTTTTT
GCGGCATTTTGCCTTCCTGTTTTTGCTCACCCAGAAACGCTGGTGAAAGTAAAAGATGCTGAAGATCAG

Determination of Flux Control Coefficients from double modulation experiments

The data from Figures 3, 4 and 9 were used to estimate the flux control coefficients of several translation factors. To accomplish this, data for each double modulation were fit to the function:

$$J = \frac{J_{max} \cdot x^n \cdot y^n}{\left(a^n + x^n\right)\cdot\left(b^n + y^n\right)} \tag{1}$$

where J is the flux, x and y are the two modulated factors, and J_{max}, $a,$ and b are two fitting parameters. When properly fit, this function approximates the data from each double modulation experiment, but no special meaning is assigned to it or its parameter values. Instead, we use it simply to estimate the value of the control coefficients, which are partial derivatives of this function. This provides a more robust estimate than simply calculating the slope of the curve by using the data (which would form a crude way of applying finite differences). Recalling that a flux control coefficients of factors x and y are expressed as

$$\begin{aligned} C_x^J &= \frac{dJ/J}{dx/x}, \\ C_y^J &= \frac{dJ/J}{dy/y}, \end{aligned} \tag{2}$$

then using (1) and (2) we obtain:

$$\begin{aligned} C_x^J &= \frac{n \cdot a^n}{\left(a^n + x^n\right)}, \\ C_y^J &= \frac{n \cdot b^n}{\left(b^n + y^n\right)}. \end{aligned} \tag{3}$$

We can therefore estimate the flux control coefficients of each factor involved in the dual modulations, which we do for the reference state (100% of each factor, corresponding to the wildtype).

eIF1 / eIF5

Data relating to Figure 2C and Supporting Fig. 2C.

Best fit parameters:

J_{max}=*101.549* *a=33.7673* *b=29.5722* *n=4.14055*

$C_{eIF1}^J = 0.046, C_{eEIF5}^J = 0.027$

eIF1 / Pab1

Data relating to Figure 2D and Supporting Fig. 2D.

Best fit parameters:

J_{max}=101.323   *a=22.9375*   *b=37.034*   *n=3.96484*

$C^{J}_{eIF1} = 0.016, C^{J}_{Pab1} = 0.076$

eEF1A / eIF4E

Data relating to Figure 5A, C.

Best fit parameters:

J_{max}=663.637   *a=1139.38*   *b=26.7552*   *n=0.529134*

$C^{J}_{eEF1A} = 0.41, C^{J}_{eIF4E} = 0.18$

eEF1A / eRF1

Data relating to Figure 5E, G.

Best fit parameters:

J_{max}=106.563   *a=34.2909*   *b=22.8072*   *n=2.34329*

$C^{J}_{eEF1A} = 0.18, C^{J}_{eRF1} = 0.071$

Summary

**Factor**	**Flux control coefficient**
eIF1	0.031*
eIF5	0.027
Pab1	0.076
eEF1A	0.30*
eIF4E	0.18
eRF1	0.071

* These values obtained as average of the two independent determinations

Supplementary Table 1
***S.cerevisiae* strain construction to top up (enhance) expression of selected genes**

**Genes of translation factors with promoter substitutions:**	**Top-up for P_{tetO7} promoter**		**Top-up for P_{CTR1}**
	integrated into *lys2* locus (with *BLE* marker)	on a *URA3* plasmid	integrated into *can1* locus (with *HIS5* marker)
P_{tetO7}*SUI1* P_{CuRE3}*TIF5*	P_{HYP2}-21utr*SUI1*	P_{LEU4} *SUI1*	P_{HYP2}*TIF5*
			none
		none	P_{HYP2}*TIF5*
			none
P_{tetO7}*SUI1* P_{CuRE4}*PAB1*	P_{EFT1}-21utr*SUI1*	*none*	P_{HYP2}*PAB1*
			none
	P_{HYP2}-21utr*SUI1*	*none*	P_{HYP2}*PAB1*
			none
	none	P_{TRP1}*SUI1*	P_{HYP2}*PAB1*
			none
	P_{TEF2} promoter substituted with:		
P_{tetO7}*TEF1* P_{CuRE3}*SUP45*	P_{HYP2} promoter with *HIS5* marker- P_{HYP2}::*HIS5TEF2*	none	none
P_{tetO7}*TEF1* P_{CuRE3}*CDC33*	P_{HYP2} promoter, *HIS5* marker removed - P_{HYP2}*TEF2*	P_{DED1}utr*TEF2*	P_{HYP2}*CDC33*
		none	
	P_{HYP2} promoter with *HIS5* marker- P_{HYP2}::*HIS5TEF2*	P_{DED1}utr*TEF2*	none
		none	
